# Microalgae Applications in the Agricultural and Food Sector: Towards a Sustainable Future

**DOI:** 10.3390/molecules31030457

**Published:** 2026-01-28

**Authors:** Emily Radican, Yangchao Luo, Zhenlei Xiao

**Affiliations:** Department of Nutritional Sciences, University of Connecticut, Storrs, CT 06269, USA

**Keywords:** microalgae, conventional agriculture, sustainability, alternative protein

## Abstract

The global population faces increasing demands for sustainable initiatives due to industrialized agriculture. To meet the demand for protein-rich foods, innovative practices must be implemented. Conventional agricultural systems face significant challenges, including soil degradation, biodiversity loss, nutrient depletion, air pollution, and degraded water quality. Additionally, conventional agriculture affects the environment due to unsustainable farming practices utilizing chemical fertilizers, pesticides, and herbicides. These practices contribute to the accumulation of greenhouse gases and carbon emissions, which negatively affect air and water quality. Agricultural yield is declining, reducing the availability of foods, and further increasing food insecurity through increased costs. Microalgae, a unicellular organism with adaptive capabilities for carbon sequestration, offers a beneficial shift from conventional agriculture. Microalgae provide low-impact environmental alternatives to the agricultural sector, promote energy conservation, and synthesize health-promoting biomolecules, such as antioxidants, pigments, essential fatty acids, polysaccharides, and protein. This review evaluates the potentials of microalgal biomass for sustainable food applications, highlighting its role in strengthening microalgae as a biorefinery and alleviating the environmental and ecological burdens of traditional farming.

## 1. Introduction

Rising global populations have destabilized our food systems [1], leading to increased global food insecurity and demands for protein-rich sources of food [2], further contributing to unsustainable farming practices [3]. These unsustainable practices are increased by resource inefficiency, particularly freshwater and groundwater resources, accounting for almost one-half of the global water footprint in food production [4]. Global food insecurity is projected to affect more than one billion people, due to significant disruptions within the global food supply chain [5]. A circular bioeconomy, such as microalgae production, presents a promising solution that will benefit the agricultural and food sector.

Importantly, long-term food insecurity contributes to malnutrition, as a result of increased nutritional deficiencies [6]. Food-insecure households are less likely to eat nutrient-dense foods due to social stigma [7]. Malnutrition impairs cognitive ability and long-term productivity [8]. Children, a vulnerable population susceptible to malnutrition [6], account for almost one-half of all deaths under 5 years old, according to the World Health Organization [9]. Poor diet quality contributes to the development of chronic conditions, such as cardiovascular disease and non-alcoholic fatty liver disease [8]. In 2024, a United States cohort study of 57,000 participants, published in the Journal of American Medical Association, highlighted an inverse relationship between life expectancy and food insecurity [10]. As of 2019, obesity combined with malnutrition incurs trillions of health-care costs per year, placing a large economic burden on the public [11]. Greater accessibility to sustainably sourced nutrient-rich compounds, such as those from microalgae, may improve nutritional access.

Microalgae are unicellular microorganisms [12], classified as either eukaryotic or prokaryotic [13]. Adaptive mechanisms enable microalgae to survive under suboptimal circumstances. Increased stress resilience produces beneficial bioactive compounds, such as antioxidants and polyphenols, in response to abiotic stressors. These secondary metabolites provide protection against damage from free radicals and reactive oxygen species. Microalgae also produce a high proportion of complete proteins, up to 70% depending on the strain, along with beneficial polysaccharides and essential fatty acids [14], providing complementary nutrition to animal-based food products. Microalgae provide micronutrients such as iron, calcium, zinc, magnesium, molybdenum, B-12, folate, and certain fat-soluble vitamins [15]. Microalgal biomass offers bioactive compounds that support immune function as a functional food ingredient. Beyond nutritional benefits, microalgae cultivation represents a sustainable alternative to conventional agriculture and addresses some key environmental challenges. This review evaluates microalgae’s potential in the agricultural and food sector to mitigate challenges related to resource depletion, livestock emission production, and food options.

## 2. Environmental Challenges in Conventional Agriculture

Inefficient resource use continues to degrade ecosystems and generate pollutants. Arable land is declining [16], water quality is deteriorating [17], soil fertility is decreasing [16], and climate instability is altering seasonal patterns, reducing agricultural productivity and predictability. Environmental detriments decline crop yields, increase production costs, and raise the consumer financial burden [18]. Water, an indispensable natural resource, is a significant driver of production costs [4]. In contrast, microalgae cultivation offers a promising solution through resource sparing effects such as carbon sequestration, reduced use of arable land, and improved water conservation [19].

Beyond inefficient resource use, crop burning, a cost-effective method for rapid land clearing, releases harmful airborne residues and pollutants [20]. Impacts commonly seen from chemical pesticide use have demonstrated correlations with adverse health effects on humans and wildlife. Chemical fertilizers seep into soil, groundwater, and nearby habitats as residual runoff accelerates the negative ecological footprint [21], increasing safety concerns [22]. Glyphosate, a common herbicide used in traditional farming, accumulates in groundwater after repeated exposure, potentially contaminating local aquatic and human ecosystems [23]. Additionally, fertilizer applications contribute to increased pollution and greenhouse gas (GHG) emissions through crop burning and the release of organic and inorganic particulates, ultimately compromising air quality [20].

Microalgae improve soil fertility due to nitrogen- and phosphorus-rich biomass, reducing the need for conventional fertilizers. *Chlorella* sp. and *Anabaena* sp. effectively serve as biofertilizers for terrestrial plants, increasing root weight and the number of leaves in cucumber seedlings. Furthermore, Chinese cabbage exhibited similar effects to conventional fertilizers, producing twice as much fresh weight, and increased protein content compared to the control [24]. Microalgal-based biofertilizers improve overall crop yield, but careful optimization based on strain and terrestrial species must be investigated to produce a valuable, cost-effective product.

Rapid land clearing and pesticide use result in evident effects within soil systems. Soil degradation occurring from mono-cropping practices, insufficient crop rotation, and industrialized agricultural machinery depletes nutrients and accelerates biodiversity loss. Soil compression from heavy machinery disrupts the soil biome, also affecting soil fertility [21]. This has resulted in the loss of nearly one-third of globally cultivated land, which is now deteriorated and unsuitable for agricultural purposes. Conservation methods enacted to protect arable land may support long-term agricultural production [16].

Unsustainable practices promote increased GHG emissions globally. In Thailand, a comparative study between traditional and organic farming increased GHG emissions, catalyzed by significant use of chemical fertilizers [25]. GHG emissions are the largest by-product of fossil fuel energy consumption [26], producing harmful methane and nitrogen by-products, such as nitrates [25]. Elsewhere, biodiversity loss in the Amazon increases as small-scale operations employ slash-and-burn agricultural methods, resulting in natural habitat displacement and ecosystem disruption [27]. Implementing sustainable agricultural practices is necessary to re-establish rich biodiversity for habitat reintegration [21].

**Figure 1:**
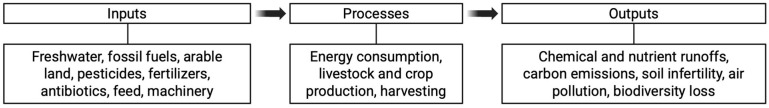
Flowchart indicating taxing inputs into conventional agriculture systems with yields and outcomes to assess linear relationships.

Additionally, present agricultural methods promote significant environmental pollution, contributing to just under one-quarter of global GHG emissions, increasing biodiversity loss [28]. Due to transportation, conventional farming generates twice the amount of carbon emissions compared to local products, with one-half of these emissions attributing to synthetic fertilizer treatments [29]. Carbon and GHG emissions increase when livestock feed is factored [30]. These current trends highlight an urgent need for sustainable practices, in order to decrease air and environmental pollution, increase biodiversity, and improve resource and energy efficiency [Figure 1]. Collectively, these deficits posit microalgae as a sustainable microorganism with diverse cultivation methods to mitigate several challenges [19,31], including resource depletion and environmental deficits through upstream applications to benefit global food security.

## 3. Microalgae Applications Within the Livestock Sector

### 3.1. Environmental Impacts of Livestock

Due to their significant contribution to GHG emissions, cattle require comprehensive environmental by-product assessments [32,33,34,35,36]. Ruminant livestock produce methane through enteric fermentation. Methane contributes largely to GHG emissions, amplifying the sector’s impact in climate change [32,33]. In South Korea, broiler production results in large volumes of GHG emissions; notably, feed production encompasses 56.8% of its total emissions. South Korea has appraised its livestock numbers at 214 million as of 2024, many of which are ruminant livestock, and the future hope within the country is to reduce carbon emissions from 21 million tons last measured in 2018 [36].

Notably, livestock contribution to global NH_3_ emissions increases in warmer climates [37]. The beef industry is a crucial component as a food source, but it comes with considerable ecological costs [32]. Supplementing 0.2% *Asparagopsis taxiformis*, a strain of red algae, reduced methane production from ruminant cattle by nearly 100%, promoting a sustainable solution to decrease air pollution [33]. In Brazil, 6% of dietary beef intake represents 50% of the country’s emissions and 33% of its water footprint [38]. By 2065, almost two-thirds of the global population will live in water scarce areas, partly due to the cattle industry and reliance on freshwater sources [34]. The beef industry requires water consumption of nearly one-third of global water use, putting agricultural production at further risk [38]. Additionally, microalgae supplementation within the livestock sector has elucidated certain health benefits, especially in broiler chickens. While ruminant livestock are the main producers of GHG emissions, poultry also plays a role. Current research highlights the potential for integrating microalgae into scalable poultry systems, providing both nutritional and environmental benefits.

### 3.2. Bioactive Compounds of Microalgae for Poultry Health

Beyond environmental benefits, microalgae influence host immunity. Immune modulation involves the reduction in pro-inflammatory cytokines and reactive oxygen species, both of which contribute to cellular and tissue damage. Bioactive compounds in microalgae, notably, sulfated polysaccharides, may contribute immunomodulating properties to the host [39]. *Spirulina* supplementation improved immune regulation in quail eggs, reducing the responsiveness of a stress gene, as well as HSP70 and IFN-γ levels, under heat stress conditions, indicating a more adaptive immune response. Mitigation of early stress responses are particularly relevant in poultry production to prevent dysfunctional growth and immune performance [40].

Beyond polysaccharides, phytochemicals in *D. salina*, including carotenoids such as β-carotene, astaxanthin, zeaxanthin, and lutein, contribute to immune regulation through their antioxidant capacity. These compounds produce beneficial effects seen in lipid profiles, egg production, and feed conversion ratios at supplementation of 0.5 g/kg of unsaturated fatty acids [41], attributed to the prevention of oxidation. Comparable outcomes have been observed in quail diets supplemented with *Spirulina,* with decreased LDL and increased HDL cholesterol occurring concurrently with increased antioxidant delivery and immune health [42]. Importantly, enhanced immune regulation is linked to integrity of the gastrointestinal tract in humans and animals [43].

### 3.3. Nutraceutical Effects of Microalgae on Metabolic Health in Poultry

In conjunction with immune function, metabolic health and growth performance play a crucial role. Metabolic health and body composition in broiler chickens are determined through multiple growth dynamics and nutrient utilization. These parameters are often assessed through feather development, breast muscles, and maturation rates. Female birds express increased lipid accumulation earlier than male birds, due to anatomical and reproductive differences [44]. Nutrient availability and utilization have direct effects on productivity and meat quality.

Microalgae have demonstrated the potential to favorably influence growth performance and meat quality in broiler chickens due to their essential fatty acid profile, mineral, antioxidant, and antimicrobial contents. Growth performance enhancements appear to be strain- and dose-dependent. Supplementation of *Chlorella vulgaris* and *Amphora coffeaformis* increased growth performance, whereas no changes were observed with *Spirulina* supplementation, and no improvements occurred in any of the tested strains related to the feed conversion ratio. Conversely, higher levels of superoxide dismutase were observed, indicating reduced lipid peroxidation and improved overall meat quality regardless of growth rate [45]. Additionally, antioxidant levels were significantly increased during limited nitrogen cultivation, prompting antioxidant synthesis to mitigate oxidative stress [46]. Stress responses influence antioxidant activity and protein, which is an essential macronutrient which supports growth and development by providing essential amino acids for protein synthesis.

Protein availability remains a critical point in growth and development. Soybean meal has a crude protein content of 40–48% and is a preferred choice for broiler diets to meet protein demands during early growth periods [47]. *Spirulina* contains up to 70% protein by dry weight and provides essential amino acids, including methionine and lysine. Dietary inclusion of *Spirulina* supplementation ranging from 3 to 12% exhibited improvements in egg quality parameters such as Haugh units, yolk pigmentation, shell thickness, and shell strength, due to high-quality essential amino acids [48]. These findings indicate *Spirulina* as a complementary protein source rather than a replacement for soybean meal [49].

Importantly, protein supplementation has strain-dependent outcomes related to biomass composition. *Chlorella vulgaris* supplementation at 5% was ineffective at increasing growth performance. However, a combination treatment of *C. vulgaris*, and *A. coffeaformis* at 1% significantly increased body weight and microbial diversity in broiler chickens, suggesting a synergistic effect [50]. *Spirulina* supplementation at 0.3% improved body weight and daily feed intake with later stage improvements in feed conversion ratio, egg production, and egg weight [51], enhancing overall productivity. These effects are likely attributed to the delivery of essential amino acids from microalgal biomass, as summarized in Table 1. Outside of microalgal protein-rich biomass, omega-3 (ω-3) and omega-6 (ω-6) fatty acids are present as well, benefitting metabolic health, immune function, and neural development in poultry.

### 3.4. Microalgae-Mediated Gastrointestinal Health and Immune Regulation in Poultry

Gastrointestinal health plays a significant role in nervous system regulation and overall immune health. *Chlorella vulgaris* contains fiber-rich biomass that support pre- and probiotic functions, improving microbial diversity and resulting in lower inflammation throughout the gastrointestinal tract [52]. Broilers have a shorter transit time through the gastrointestinal tract compared to other livestock. The integrity of the epithelial barrier, and microbial diversity are critical to maintaining proper immune responsiveness, including central nervous system preparedness against stress and inflammation. Potentially, the gut–brain axis in broilers stimulates stress and behavioral changes through cortisol, cytokine, and neurotransmitter release, ultimately affecting the epithelial integrity in the gastrointestinal tract [43], compromising immune function.

Additionally, *Spirulina* supplementation in broiler diets mitigated heat stress and immune response. *Oscillospira*, a commensal bacterium, was subsequently lower in broilers that experienced heat stress, due to poor recovery. *Spirulina* supplementation improved gastrointestinal integrity-associated genes [53], indicating beneficial effects on gastrointestinal-related immune function. These findings elucidate microalgae as a potential broiler diet addition to improving humoral immunity.

Short chain fatty acids, a by-product of colonic bacterial fermentation, assist in immune health and provide essential energy. Mishra et al. assessed microalgae supplementation and found no beneficial impact to SCFA production; however, differential microbiota populations were observed. The expressed populations correlated with increased nutritional bioavailability in broilers [54], enhancing nutrient utilization. Additionally, prevalent methods for immune regulation in livestock include antibiotic supplementation in animal feed to lower the risk of pathogenic diseases. However, antibiotic resistance is an ever-growing public safety concern [55] that necessitates further investigation due to its potential to increase contamination of antibiotic-resistant genes that infect humans and ecosystems.

### 3.5. Antibacterial Potential of Microalgae

Finally, microalgae are positioned as a potential solution to antibiotic resistance (AR). AR is a looming threat for public health and safety due to long-term antibiotic supplementation in animal feed, which is banned in the European Union as a growth promoter [42]. The mechanism of resistance increases when AR genes are transferred across bacterial species through horizontal gene transfer [56]. According to the Centers for Disease Control (CDC), AR largely impacts animal and human populations, allowing pathogenic bacteria to proliferate, causing over one million deaths per year globally [57]. Poultry, specifically commercial poultry, is susceptible to AR bacterial strains, such as E. coli and Salmonella [58]. In Egypt, manure samples of poultry showed antibiotic-resistant genes present in over 40% of samples, specifically to tetracycline, a broad-spectrum antibiotic, through terrestrial and aquatic environments [59]. AR is a growing concern that underscores the need to identify alternatives to antibiotic-supplemented animal feed.

Various studies have highlighted the antibacterial and antimicrobial applications of microalgae. Microalgae–bacterial granular sludge complex exhibited the potential to treat antibiotic wastewater, which is important for the prevention of eutrophication. Furthermore, microalgae assisted AR gene biocontrol through metabolic pathways and by decreasing bacterial proliferation. These findings present opportunities for microalgae as a potential treatment for antibiotic-rich wastewater [51]. Additionally, *Spirulina* extract inhibited pathogenic proliferation, positing it as a potential alternative supplement to antibiotic-rich poultry feed [42]. Given the rise in AR, the antimicrobial compounds in microalgae, such as phenols, are a valuable feed additive to supplement antibiotic use in livestock [59]. Further assessments of *C. sorokiniana*, *Chlorella* sp. and *Scenedesmus* sp. included phenolic content and antimicrobial compounds effects in 18 bacterial strains. *Chlorella* sp. exhibited the greatest antimicrobial effects, and all strains exhibited no cytotoxic effects with high bactericidal efficiency [60], meaning no cellular toxicity occurred. Controlled environment agriculture, specifically microalgae cultivation in closed photobioreactors or indoor systems, may present a lower risk for AR.

## 4. Upstream Microalgae Production

### 4.1. Microalgae Cultivation Systems

Downstream applications must be supported through scalable cultivation of microalgae. Controlled environment agriculture (CEA), specifically microalgae cultivation, may be a useful tool to improve climate change and reduce food insecurity, while supporting economic growth [61]. Broadly, CEA includes vertical farming, hydroponics, aeroponics, and aquaponics to control factors such as ambient temperature, lighting, and climate to optimize plant biomass and increase yield [62]. Importantly, microalgae cultivation is diverse and controllable, offering a promising potential for product yield. Table 2 compares the resource use and environmental impacts in traditional agriculture and microalgae cultivation. Microalgae cultivation is one subset of CEA technology, encompassing both eukaryotic microalgae and prokaryotic cyanobacterium that are capable of carbon fixation through photosynthesis. Compared to terrestrial plants, microalgae exhibit 10–50 times more carbon sequestration efficiency [63].

**Figure 2:**
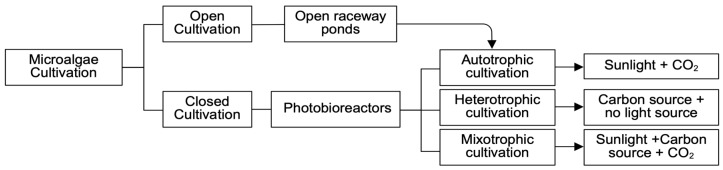
Comparison of energy requirements for microalgae cultivation methods.

Additionally, microalgae utilize energy conservation systems, while synthesizing beneficial metabolites and biomolecules [64,65], including pigments which may offer cardiovascular and neuroprotective effects. Enhanced pigment and rapid biomass accumulation indicate greater photosynthetic efficiency [66], where *D. suspicatus* produced 46% higher carotenoid content in a photobioreactor due to broth circulation [67]. Microalgal biomass is influenced by location and light source, with the latter being the most pertinent factor. Microalgal species, even within the same family, require different growth conditions to accumulate metabolites and biomass efficiently [64]. Cultivation is conducted through two main pathways: open systems in raceways ponds, or closed systems in photobioreactors. Photobioreactors are the industry standard and involve controlled cultivation parameters with low contamination risk [19,31]. Additionally, photobioreactors have optimal circulation of required nutrients, improving yield compared to open raceway ponds [31]. Figure 2 outlines the two main approaches for microalgae cultivation through either open or closed systems, and, subsequently, energy consumption.

While an open system favors cost-effective production, the photobioreactors involved added gas transfer, increased operation costs, with reduced contamination risk, as depicted in Table 3. Production values include biomass productivity, COD removal, and photosynthetic efficiency. COD, or chemical oxygen demand, indicates organic loading and contamination potential; higher COD values correspond to an increased risk of microbial contamination. Closed system cultivation exhibited increased biomass productivity, COD removal, and photosynthetic efficiency, compared to an open system cultivation with increased productivity [68]. By far, the discussion of microalgae highlights many benefits including synthesis of bioactive compounds and secondary metabolites, improved immune modulation, increased antimicrobial properties, and livestock and environmental benefits. Alternatively, we must evaluate microalgal consumption safety due to their ability to host contaminants and accumulate toxins.

### 4.2. Nutrient-Rich Effluents for Microalgae Cultivation and Biofuel Production

Beyond traditional cultivation methods [Figure 2], nutrient-rich effluents such as anaerobic digestate (AD), whey wastewater, and other industrial waste streams have emerged as substitute growth media for microalgae cultivation. These effluents may be viable alternatives to conventional nitrogen-based fertilizers [69], and reduce overall production costs. Additionally, *C. vulgaris* acted as an effective bioremediation tool by removing nitrates, phosphates, and other harmful environmental contaminants in treated sewage wastewater [70].

Partial AD substitution of cultivation media is an emerging method due to its high ammonium content, providing a rich nitrogen source for microalgal growth [71]. Murdoch University, Australia, assessed microalgal growth potential in AD pig effluent, using *Chlorella* sp., *Scenedesmus* and pennate diatom. By adding CO_2_ to the effluent culture, ammonia is converted to ammonium, stabilizing the culture and mitigating nitrogen toxicity [72]. Cattle effluent at 1 g/L resulted in reduced carbohydrate content in microalgae, resulting in higher protein concentrations compared to the control, improving protein production in 100 L applications [73], thus highlighting relevant industry scale potential. These animal waste effluents provide high-value nutrients to microalgae cultivation; additionally, they recycle nutrients back into the soil, increasing soil fertility [74]. These findings support valorization of nutrient-rich effluents and wastewater to increase microalgae value with minimal biomass waste. However, further optimization is necessary due to variability among effluent compositions.

Microalgae outdoor cultivation in whey permeate, a dairy wastewater rich in phosphorus, nitrogen, and carbon, efficiently increased overall biomass in a polyculture compared to a glucose control. This mixotrophic cultivation highlights a year-round application for whey wastewater remediation and improved protein productivity [75]. Whey wastewater by-product rises each year with an increased demand of dairy products. Previously, improper removal and lack of pre-treatment of wastewater led to eutrophication, or excessive nutrient loading into bodies of water, bringing harm to aquatic life [76]. Whey wastewater cultivation offers a sustainable valorization pathway for nutrient recovery. Nutrient-rich effluents highlight potential cultivation alternatives, decreasing production costs and improving environmental load.

Nutrient-rich wastewater containing carbon, nitrogen, and phosphorus are nutrients required for microalgal growth, presenting a promising approach to reduce biofuel production costs [77]. Additionally, further cost-reducing measures include bacterial co-cultures with symbiotic nutrient-exchange relationships with bacterial-produced ammonium, and microalgal-produced carbon, resulting in greater biomass productivity, such as those seen in *Chlamydomonas reinhardtii* [78]. Microalgae-based biofuels are a rapidly expanding area of research aimed to provide cleaner and more sustainable energy sources. Biofuel production, particularly microalgal-based biodiesel, focuses on the lipid content within the biomass. A study conducted at the College of Environmental Science and Engineering in China utilized acidified starch wastewater with AD to demonstrate lipid bioaccumulation [77].

Many wastewater facilities contain contaminants such as pesticides, hormones, PFAS, and AR bacteria, with higher than acceptable contaminant values, indicating potential damage to vertebrates and invertebrates [79]. Due to these biological and chemical hazards, barriers exist regarding using wastewater effluents outside of fertilizers and biofuels, indicating the necessity of establishing improved pre-treatment and safety monitoring systems. Microalgal biofuel production lowers GHG emissions, decreasing the effects of climate change [80] compared to conventional agriculture. With optimized cultivation, microalgae-derived lipids may compete with food-sourced lipids for bioenergy. This approach strengthens the circular bioeconomy by reducing production costs and enhancing microalgal biomass value in bioenergy beyond the food sector [77].

### 4.3. Microalgae Cultivation Risks and Mitigation Techniques

Heavy metals accumulate in bodily tissues and act as toxins to multiple organ systems. Mercury, lead, thallium, cadmium, molybdenum, and cobalt, which are examples of heavy metals, have significant impacts on cardiovascular, neural, hepatic, renal, and endocrine systems, posing carcinogenic risks [81]. Certain heavy metal exposures occur through skin contact or inhalation, and do not require ingestion of heavy metals for exposure to occur [82]. Due to the role of microalgae in environmental sequestration of heavy metals, different pathways are involved in cellular response based on the species and metals present [83]. The bioremediation potential of microalgae occurs due to passive biosorption into the cell, followed by active diffusion in bioaccumulation intracellularly [84]. *A. coffaeiformis* exhibited a 55% decreased growth rate post chromium exposure. *D. salina* decreased its growth rate by 60%, and *Navicula salinicola* decreased it by 55%, post cadmium exposure. Both *A. coffaeiformis* and *D. salina* exhibited higher expression in chelator genes in the presence of lead and cadmium treatments. Due to their binding affinity, microalgae possess bioremediation potential [82], increasing speculation on safe cultivation techniques to prevent heavy metal accumulation. Notably, certain strains with higher sensitivity to heavy metals may result in decreased production of biomass [85], whereas certain strains have developed evolutionary mechanisms to resist heavy metal toxicity through counter production within the cells antioxidant systems [84].

Current industry interventions to mitigate microbial control include membrane filtration and pH control. A 2 μm membrane filter and pH adjustment were employed in *Spirulina* co-culturing of *Chlorella* species to prevent unwanted contamination [86]. Pleissner et al. reported that a pH reduction to 3.5 was beneficial in killing *Vampirovibrio chlorellavorus*, a bacterium that prey on algae. Alternatively, increasing the pH to greater than 11 had the same effect on protozoan species, leading to a growth decline [87]. While these methods are effective at microbial control, strain specificity may pose a limitation if pH is outside the optimal growth conditions. Aside from intervention methods, quantification of toxins, especially microcystins, a by-product of many cyanobacteria, are crucial to assess product safety. Microcystins, a known human carcinogen, present toxicity to renal, hepatic, and neural systems in humans and animals. [88]. Dose-dependent death occurs in animals exposed to high quantities of microcystin populations [89], where the FDA released warnings related to exposure and daily dosage limits. Heavy metals, toxins, and microbial contaminants are potential effects of open microalgae cultivation, highlighting the benefits of a closed system to improve microalgal biomass safety in food applications. Addressing these risks through controlled cultivation involving strain selection and monitored cultivation is essential for microalgae’s application within the food and feed sector.

## 5. Downstream Microalgae Applications: The Food Industry

### 5.1. Nutraceutical and Food Applications of Microalgae

Microalgae produce functional food ingredients, expanding their value in the algal chain. Certain strains have been reported to contain up to 50–70% of their dried biomass as complete protein, though protein content widely varies between species, cultivation conditions, and harvesting methods. Microalgal proteins provide a promising alternative source in the plant-based food industry, but consistency within industrial scale may remain a challenge [90,91,92]. *Spirulina* fortified plant-based crostini’s by-partial substitution of flour, creating a protein-rich product, with some minor limitations including sensory attributes, and unwanted green coloring in the final product [93]. *Spirulina* protein isolates exhibited high foaming capacity efficiency and high zeta potential, indicating great stability within colloidal dispersions, and offering a promising alternative to soy protein isolates [94] due to high-value amino acids. Although soy protein isolates are plant-based, increased quantity of plant-based protein options improves the market value of plant-based products.

Besides functional protein alternatives, microalgae produce and release volatile organic compounds (VOC). Microalgal strains that contain sulfur-based compounds mimic the aroma and taste of seafood for plant-based applications. This offers a sustainable alternative to overfishing [95]; however, improvements on sensory profile need to be addressed to ensure a high-value, high-quality product. Generally, microalgae-emitted VOCs [95] and certain aromatic compounds [96] are associated with lower consumer preference [93]. Prior to developing a wall material encapsulation system, identifying a VOC profile is beneficial in determining the materials to encapsulate, and the potential application. Four strains were profiled, namely *Spirulina*, *C. pyrenoidosa*, *C. reinhardtii*., *H. pluvialis*, detecting ketones, aldehydes, esters, phenols, hydrocarbons, and sulfur-based compounds. Methanethiol and dimethyl trisulfide are sulfur-based compounds that are potent and generally responsible for unwanted aromas and flavors in microalgal biomass [96].

Commonly proposed methods for flavor masking include microencapsulation, involving crosslinking biopolymers to form a wall material [97], effectively trapping undesirable flavors and improving bioactive compound stability through functional group interactions [96]. Homogenous microcapsules are produced through spray-drying [97]. *Spirulina* is spray-dried with maltodextrin, a carrier starch, and citric acid, and then crosslinked to form an effective wall material, improving the sensory profile in tested yogurt products [98]. Freeze drying yielded greater protein solubility and emulsion capacity compared to spray-dried *C. vulgaris*. Greater odor masking occurred in freeze drying, compared to spray-drying, where no wall materials were applied [99]. These applications involving fortification and plant-based analogs can be further improved through enhanced processing techniques.

Optimized processing techniques improve harvesting for functional biomolecules [100]. High-pressure processing techniques exhibit optimal microalgae protein extraction in applications as gelling agents, emulsifiers, or quality protein replacements [92], increasing meat analog applications. Cell wall disruption under high-pressure mechanical processing allows increased utilization of intracellular components [101], such as plant-based meat extrudates. Carotenoids, an antioxidant pigment in plant-based sources including microalgae, can be used as a food colorant while offering health-promoting benefits. *Chlorella* exhibited increased antioxidant recovery yield due to the pressurized liquid extraction (PLE) technique [102], improving its nutraceutical value [102,103].

Additionally, mechanical processing techniques of microalgae positively alter rheological properties to mimic the mouthfeel, texture, and appearance of animal-based meat. Meat substitutes or analogs aim to mimic characteristics of animal-based meat products, while providing suitable nutrition for a plant-based diet [104]. Microalgal proteins tend to aggregate due to their low isoelectric point, underscoring a need for optimized processing conditions to improve protein texture, and enhanced emulsification for food application. Low acyl gellan gum improves microalgae protein stability due to greater hydrogen bonding, resulting in lower zeta potential [105], which may reduce its stability within colloidal dispersions, limiting applications. *Chlorella* species promote gelation properties through fibrous network development during homogenization due to protein fiber elongation, improving product texture [91]. Optimized microalgae processing techniques improve food and nutraceutical applications.

Agitated thin film drying resulted in the highest functional application properties; however, it lacks widespread use in the food industry related to microalgae, presenting a promising method for energy conservation to deliver a stable product [99]. Coacervation, a phase separation of two immiscible liquids, promotes the assembly of microcapsules, improving the sensory profile of *Chlorella* in a pear snack application [97]. Ionic gelation involves electrostatic interactions and crosslinking, where alginate and calcium chloride are commonly used materials. With external gelation, greater crosslinking reduced diffusion rates, increasing encapsulation efficiency and subsequent flavor masking [106]. This research underscores the importance of compound profiling for aroma and flavor assessment for optimized flavor masking to improve sensory profiles in microalgae.

**Figure 3:**
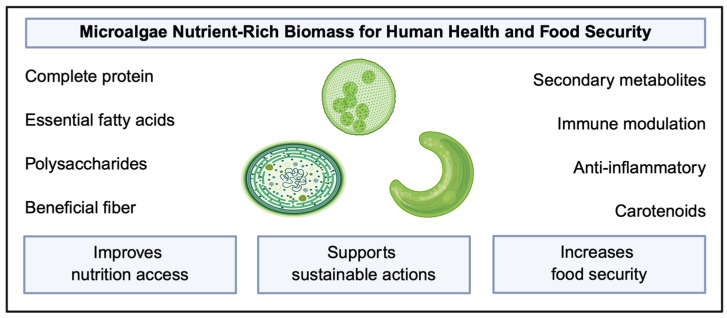
Microalgae-derived bioactive compounds and their benefits for human health.

Aside from functional metabolites and food products, microalgae contain nutrient-dense biomolecules with high levels of beneficial polysaccharides and essential fatty acids [Figure 3] [91,107,108]. Compared to conventional aquatic farming, microalgae cultivation presents a more sustainable alternative. Certain strains of microalgae contain essential fatty acids in the form of eicosapentaenoic acid (EPA) and docosahexaenoic acid (DHA), delivering a highly bioavailable, sustainable EPA:DHA alternative supplement [19]. Due to prominent levels of essential fatty acids [108], microalgae offer a comparable alternative to fish oil supplementation [65]. Notably, *Chlorella*, *Spirulina*, and *Synechococcus* contain sulfated polysaccharides shown to lower inflammatory protein markers linked to inflammatory bowel disease [109]. Additionally, fiber content in microalgae offers a beneficial food source for colonic bacteria. Colonic bacteria utilize fiber as a food source, producing secondary metabolites, such as short-chain fatty acids (propionate, butyrate, acetate), which assist in modulation of the gastrointestinal tract, potentially lowering inflammation [65]. Given that a large subset of the population does not meet the RDA for fiber intake, a diet high in fiber may ameliorate risks associated with colon cancer [110]. Microalgae-derived bioactive compounds are present as a nutrient-dense food source, including high-fiber supplementation.

### 5.2. Nutritional Comparison and Bioavailability of Spirulina and Common Dietary Foods

Microalgae offer a suitable plant-based protein source among other nutrients and may offer a promising role in the food sector for future sustainable food systems. *Spirulina*, among other microalgae, have obtained ‘generally recognized as safe’ (GRAS) status, granted through the FDA, containing high protein and mineral content [104], compared to lentils, beef, or haddock [111]. *Spirulina* is one of the most widely used microalgal species in the food sector, making it an appropriate choice for a comparison below [Table 4] [112].

While optimization of microalgae production includes increased costs, including cultivation and processing to improve biomass yield, as well as product formulation, there is evidence of a promising nutrient source [113]. Notably, bioavailability must be assessed due to reported content that is not reflective of exogenous uptake. Although lentils contain plant-based protein, and other minerals, the bioavailability of plant-based iron, such as non-heme iron, is much lower (1–10% compared to heme iron, 25–30% from animal products) [114]. Alternatively, *Spirulina*-fortified rat food exhibited increased iron uptake and antioxidant activity, and improved protein malabsorption. These results demonstrates *Spirulina’s* ability to lower oxidative stress while increasing protein and iron serum values [115]. Importantly, plant-based dieters are exposed to lower bioavailable iron sources, where microalgae may prove to be beneficial in improving serum values and iron stores within the body.

Bioavailability of plant-based foods such as legumes, grains, fruits, and vegetables are affected by anti-nutritional factors, such as lectins, phytates, and oxalates. Absorption of nutrients is impacted when anti-nutritional factors bind to the nutrient and form a complex affecting active binding at the delivery site. Food preparation techniques, such as fermentation, germination, or thermal processing, reduce anti-nutritional factors, and improve bioaccessibility. Protein extraction from faba beans via the alkaline isoelectric method is effective [116], but it may yield a time-intensive process. Per 100 g, *Spirulina* has higher levels of iron, calcium, and magnesium content per edible portion compared to ground beef and haddock. Notably, 100 g is an unrealistic portion size of microalgae, whereas 20 g is a more relatable portion size, which provides 9.41 g protein, 3.2 mg iron, 41 mg calcium, and 97 mg magnesium. Ready-to-eat microalgae products would need to be prepared according to previously mentioned processing and encapsulation techniques, incurring further costs to improve sensory profile. Comparably, when eating beef or fish, minimal alterations in the protein are involved prior to ingesting. While this represents a limited comparison of commonly consumed foods, these values highlight microalgae’s potential to deliver dietary nutrients, especially in plant-based dieters.

## 6. Economic and Regulatory Considerations of Microalgae

### 6.1. Economic Scalability of Microalgae Production and Current Production

While microalgae offer numerous environmental, ecological, and food applications, their economic feasibility remains a critical consideration. As photosynthetically efficient microorganisms adaptable to diverse cultivations, microalgae benefit from closed-system cultivation due to reduced contamination risk, although at increased production costs [117]. Engineering a functional ‘hanging bag’ photobioreactor design can reduce operating costs and improve biomass productivity [118]. Production costs were significantly reduced, up to 90%, in aquaculture hatcheries, where labor and facility size contribute significantly to overall cultivation costs, meaning strategic planning is essential to keep costs low [117]. Although glucose is a common carbon source, substituting sodium acetate provides a more cost-effective alternative while supporting sustainable protein production [119]. Microalgae exhibited an 89% decrease in energy consumption compared to standard combustion-based carbon capture systems, indicating that there is significant ability for energy conservation [120]. Optimizing open-pond cultivation, including ambit temperature and mixing rates, reduced energy costs by 30% [121].

Oostlander et al. reported up to a 36% reduction in production costs when artificial light was applied, whereas Blanken et al. calculated increased production costs due to similar conditions [117,122], a discrepancy which may be related to differences in biomass productivity and light energy efficiency. While these strategies alleviate some economic burdens, the versatility of microalgae remains valuable across multiple industries. For example, biofuel production from microalgae cultivated in cheese-whey wastewater demonstrated increased lipid productivity, while reducing costs close in parity with commercial oils [123]. Conversely, Van der Strict et al. (2023) found that European consumers were willing to pay premiums for certified organic and sustainable labels [2], indicating that cost sensitivity varies across socioeconomic groups. Ultimately, an understanding of production costs is essential to advance microalgae cultivation as a green technology for the focal point of future research.

Additionally, many companies are currently producing microalgae-based products suitable for applications within the agricultural and food sectors. Table 5 highlights relevant industry examples of current products on the market. This comparison includes a limited selection of prevalent companies within the algal industry, illustrating primary functional applications of microalgae, particularly protein supplementation, antioxidant support, and omega-3 fatty acids. Table 5 focuses on a limited number of common taxa, *Spirulina*, *Chlorella*, and *D. salinaI*, where many of the downstream applications focus on nutraceutical applications. While this list is not exhaustive, it is intended to demonstrate existing commercial uses of microalgal-based products in the food sector, reflecting a potential regulatory and consumer acceptance ease. However, it does highlight a gap of strain diversity, where regulatory familiarity may be a constraint, especially since significantly more taxa are researched compared to those approved for market use. This signifies that through regulatory applications, recognized taxa may move to the market faster than those that are more obscure.

### 6.2. Regulatory Considerations of Microalgae in the Food Sector

Regulatory safeguards are essential for protecting public health, particularly for food additives without established safe consumption levels, which would undergo an extensive safety evaluation process. These evaluations assess allergenic and toxicological potential prior to approval for consumer use [112]. While both regulatory agencies prioritize consumer safety, they differ in assessment approach, timelines, and data requirements. In the United States, food ingredients undergo evaluation to obtain GRAS status, or Generally Recognized as Safe, through the Food and Drug Administration. This process involves expert assessment of safety parameters based on the intended use and exposure of an ingredient [124].

Initially, a food additive petition for GRAS status may be filed, and the FDA and U.S. Department of Agriculture assesses product relevance related to livestock products. Safety must be established, including tolerable upper intake limits, along with extensive assurances regarding the intended use of the prospective additive in food products. Once the petition is finalized, regulations are based on U.S. Code of Federal Regulations, which outlines labeling and specification requirements. After market release, the FDA periodically assesses GRAS status on the products based on current scientific opinions regarding safety [125].

Currently, several microalgal strains have achieved GRAS status, including *Spirulina*, *Chlorella protothecoides*, *Dunaliella bardawil*, and *Haematococcus pluvialis* [124], indicating that their functional components, extracts, derivatives, or whole biomass are approved for safe use in food products. As indicated in Table 6, the primary use of these microalgae strains is as nutrient-dense and functional ingredients to enhance protein content, providing antioxidants and improving overall nutritional quality within a food matrix.

Similarly, the European Parliament emphasized algae as a key sector in 2022 to advance sustainability across food, feed, pharmaceuticals, biofuels, bioplastics, cosmetics, and environmental restoration [126]. Under EU Regulation 2015/2283, novel foods, defined as those not regularly sold and consumed prior to 15 May 1997, must undergo an extensive assessment and monitoring process before being granted market authorization [127].

Since 2018, the European Food Safety Authority (EFSA) has served as the sole authority for novel food assessment in the EU, conducting comprehensive product characterization, including chemical and microbial analysis, as well as toxicity and stability testing [128]. Regarding novel food applications, EFSA follows a structured process that includes pre-submission, submission, risk assessment, and post-adoption. When all required documentation is submitted, the typical processing time for risk assessment is approximately 9 months, which may vary by case, especially if extra safety testing is needed [129]. Prior to a final assessment within the EFSA, the panel on Nutrition, Novel Foods, and Food Allergens utilizes expertise to provide an assessment of consumer safety in the proposed novel food. After a novel food has undergone the EFSA process, the EU has final authorization for novel foods for market release [130].

Key differences between these regulatory frameworks include the U.S emphasis on current scientific evidence and expert consensus to support market acceptability, compared to the EU, which involves a more centralized and precautionary approach, typically resulting in longer approval times for market release of food additives. These evolving policies and regulations present opportunities to expand international collaboration efforts to improve sustainable systems, particularly in agriculture and food production. Sustainable initiatives and incorporated safety considerations will help increase consumer acceptability of future products to support the development of a microalgal circular bioeconomy.

## 7. Future Directions

While microalgae cultivation represents a sustainable initiative within the food and feed sectors, critical research gaps remain, particularly in the development of species-specific optimized cultivation standards. Future research should prioritize integrated optimization strategies to support industrial scale-up and commercial applicability. Key research questions include how to minimize energy inputs and production costs, ensuring product safety by preventing contamination and toxin accumulation during cultivation, and which processing and formulation strategies are most effective for flavor masking and improving consumer acceptance of microalgae-based products. A targeted approach focusing on these research gaps will escalate microalgae’s further integration into the food and feed sectors to improve agricultural deficits and global food security.

## Figures and Tables

**Table 1:** Summary highlighting microalgae supplementation effects in poultry related to growth metrics.

Microalgae Strain	Supplementation	Observed Effect	Parameter Involved	Reference
*Spirulina*	0.3%	Increased body weight, egg production, and feed intake	Growth performance	[51]
*C. vulgaris*	5%, 1% with *A. coffeaformis*	5% had no effect, 1% increased body weight	Body weight, microbiome diversity	[50]
*D. salina*	0.5 g/kg	Improved lipid panel	Lipid profile, antioxidant status, prevention of oxidation	[41]
*Spirulina*	3–12%	Improved yolk color, increased shell thickness	Egg quality	[48]
*C. vulgaris*	Non-specific	Improved epithelial integrity, decreased inflammatory markers	Immune function	[52]

Note: The list is not exhaustive of all microalgae-based poultry supplementation.

**Table 2:** Qualitative comparison of traditional agriculture vs. controlled environment agriculture.

Resource	Traditional Agriculture	Microalgae Cultivation
Freshwater	Global food production equates to ~50% of water consumption; droughts	Utilizes wastewater systems, or recirculates freshwater for cultivation; resource-efficient
Arable land	~33% of arable land lost; reduced soil fertility	Minimal arable land footprint: vertical and photobioreactor systems may be used in place to preserve arable land
Pest management	Chemical pesticides, herbicides, fungicides leave residues; potential leaching	Closed systems reduce exposure and contamination
GHG emissions	Methane, nitrous oxide contribution to environment; slash-and-burn technique	High carbon fixation efficiency; 10–50 times more than terrestrial plants
Biodiversity	Declining due to climate change; monocropping; soil fertility loss	Reduced interference with habitats

**Table 3:** Differences between open and closed systems of microalgal cultivation.

Method of Cultivation	Energy Consumption	Production Costs	Productivity	Contamination Risk
Open raceway ponds	Decreased energy consumption due to ample access to sunlight	Easy design, greater production capacity	Higher production capacity if climate and other environmental factors are favorable	Increased contamination risk, heavy metals, microbes, etc.
Closed systems (PBR)	Increased energy consumption due to artificial lighting, gas transfer	Higher production costs due to cultivation chamber setup; CO_2_ transfer	Higher productivity due to controllable cultivation	Lower risk of contamination if cultivation is performed through axenic conditions

**Table 4:** Nutrient composition of Spirulina compared to common food sources (per 100 g edible portion).

Nutrient	*Spirulina platensis*	Lentil (Dry)	Beef (Ground 80% Lean)	Fish (Haddock)
Protein	47.04 g/100 g [113]	23.6 g/100 g [111]	17.5 g/100 g [111]	16.3 g/100 g [111]
Iron	16 mg/100 g [113]	7.16 mg/100 g [111]	1.96 mg/100 g [111]	0.17 mg/100 g [111]
Calcium	207 mg/100 g [113]	62 mg/100 g [111]	7 mg/100 g [111]	11 mg/100 g [111]
Magnesium	486 mg/100 g [113]	107 mg/100 g [111]	16.4 mg/100 g [111]	21.2 mg/100 g [111]

Note: Nutritional value of lentils (dry), beef (ground 80% lean), and fish (haddock) obtained from USDA agricultural website [111]. *Spirulina* nutritional content adapted from [113].

**Table 5:** Comparison of algal-based products in several companies.

Company	Species	Product Form	Application	Website
Cyanotech	*Spirulina*	Whole biomass; tablets	Immune health; dietary supplementation; high absorptive capacity	https://www.cyanotech.com/spirulina/ URL accessed on 10 January 2026
Earthrise Nutritionals	*Spirulina*	Dried whole biomass; powder; tablets	Protein smoothie; supplemental protein	https://www.earthrise.com/ URL accessed on 10 January 2026
Life Extension	*Chlorella* sp.	Tablets (500 mg)	Vegan-suitable vitamin B-12 supplementation	https://www.lifeextension.com URL accessed on 10 January 2026
AstaPure	*Haematococcus pluvialis*	Astaxanthin extract	Antioxidant supplementation and support	https://astapure.com/ URL accessed on 10 January 2026
Bluebonnet Nutrition	*Dunaliella salina*	Softgel; β-carotene extract	Eye and immune health support; antioxidant function	https://bluebonnetnutrition.com URL accessed on 10 January 2026
Algama Foods	*Chlorella* sp.	Formulated ingredient blend	Plant-based egg replacements; bakery applications	https://algamafoods.com URL accessed on 10 January 2026
Algenuity	*Chlorella vulgaris*	Whole biomass powder	Supplemental dietary protein; food and beverage applications; fiber supplement; nutraceuticals	https://algenuity.com/ URL accessed on 10 January 2026
Phycom	Algal blend	Whole biomass	Protein supplementation in meat analogs; emulsifier	https://phycom.eu/ URL accessed on 10 January 2026
Nordic Naturals	Algal blend	Algal oil	Vegan-suitable DHA supplement; Omega-3	https://www.nordic.com/ URL accessed on 10 January 2026
DSM-Firmenich	Algal blend	Algal oil	Vegan suitable DHA supplement; Omega-3	https://www.dsm-firmenich.com/en/home.html URL accessed on 10 January 2026

Note: Information is compiled from publicly available product documentation and manufacturer disclosures. The list is not exhaustive and does not imply any endorsement.

**Table 6:** Examples of current microalgae strains granted GRAS status through the FDA and their proposed and approved uses and dosages [124].

Species	Component	Common Use	Usage Restrictions	Approved Dosage	GRN No.
*Chlorella sorokiniana*	Whole biomass	Protein fortification in baked goods, nutritional protein powder supplements	n/a	5–10 mg per g	986
*Chlorella protothecoides*	Whole biomass	Flour substitute to add nutritional value to baked goods, such as but not limited to added protein content	May require color additive listing	Up to 5562 mg per day	519
*Chlorella protothecoides*	Algal oil	Cooking and baking substitution for vegetable oil	n/a	Up to 26% in baked goods and beverage bases	384
*Spirulina*	Phycocyanin	Food ingredient	Baby foods and formulas	Up to 250 mg per serving	424
*Spirulina*	Whole dried biomass	Cereals, fruit juice, grain products	Tolerable upper limits include 4132 mg/kg	Up to 3 g per serving	394
*Haematococcus pluvialis*	Astaxanthin extract	Baked goods, juices, processed foods	n/a	0.15 mg/serving	580
*Dunaliella bardawil*	Whole dried biomass	Cheese, baked goods, condiments	May require color additive listing	100 mg per kg	351
*Schizochytrium* sp.	Algal oil (>35% DHA)	Ingredient in milk and plant-based milk beverages	0.5% (*w*/*w*) in infant formula	5.8% (*w*/*w*)	934

Note: Uses and dosages reflective of data from FDA GRAS Notices (GRN). This list is not exhaustive. [n/a] is indicative of no listed taxa restrictions on the official FDA website.

## Data Availability

No new data were created or analyzed in this study.
